# A school-based rope skipping intervention for adolescents in Hong Kong: protocol of a matched-pair cluster randomized controlled trial

**DOI:** 10.1186/1471-2458-14-535

**Published:** 2014-05-30

**Authors:** Amy S Ha, Chris Lonsdale, Johan Y Y Ng, David R Lubans

**Affiliations:** 1 Department of Sports Science and Physical Education, The Chinese University of Hong Kong, Shatin, Hong Kong; 2 Institute for Positive Psychology and Education, Australian Catholic University, 25A Barker Road, Strathfield, NSW 2135, Australia; 3 Health and Physical Education Building, The University of Newcastle, Callaghan University Drive, Callaghan, NSW 2308, Australia

**Keywords:** Physical education, Intervention, Moderate-to-vigorous physical activity, Self-determination, Motivation, Multilevel modeling

## Abstract

**Background:**

Schools present venues for physical activity promotion among youth, with physical education (PE) considered the primary vehicle responsible for increasing activity levels. Yet students are not very physically active during typical school PE classes. With the aim to engage Hong Kong students in more moderate-to-vigorous physical activity (MVPA) during PE, a fitness infusion intervention using rope skipping was designed, and its effectiveness will be examined. Rope skipping was chosen because a) it provides moderate-to-high intensity physical activity; b) is inexpensive; c) students find it enjoyable; and d) is feasible in typical environments in Hong Kong, where PE classes are large in size (up to 40 students) and space available for physical activity is usually limited.

**Methods/Design:**

A matched-pair cluster randomized controlled trial was designed. Secondary school students from 24 classes (from 12 schools) will be recruited to participate in the trial. Students’ baseline MVPA will be measured during school PE. Classes will be matched according to baseline variables and one class from each pair will be randomized into the experimental group. Teachers in the experimental group will be invited to attend a teacher workshop, and will insert a 15-minute rope skipping activity in four consecutive PE lessons. Motivational factors based on self-determination theory will also be measured as secondary outcomes. The effectiveness of the intervention will be evaluated by comparing changes in the proportion of lesson time spent in MVPA from baseline to follow-up across the experimental and control groups.

**Discussion:**

Physical activity levels in PE are often very low and there is a need to identify feasible low-cost interventions that can be easily disseminated. If the results of the study suggest the intervention to be effective, it could be implemented to schools throughout Hong Kong and other cities where space is limited.

**Trail registration:**

ANZCTR: ACTRN12613000968774. Registered on 30 August 2013.

## Background

Despite the extensive physical and psychological health benefits of physical activity (PA) [1,2], many children and adolescents are not sufficiently active [3,4]. One way of promoting PA in this population is through school physical education (PE), which provides a venue for all students to engage in moderate-to-vigorous physical activity (MVPA) and accrue immediate health benefits [5]. Apart from that, another important role of PE is to provide students with knowledge, skills, and confidence to stay active throughout their lifetime [5]. Through PE, students can also acquire self-management skills, and to experience and learn about different types of games and activities [6]. Nonetheless, to counter low habitual PA levels, it has been suggested that school PE should be designed to keep students active for at least 50% of the class time [7]. Unfortunately, activity levels in PE are often very low, with students spending only 27% to 47% of their lesson time in MVPA [8]. Furthermore, results of studies conducted in Hong Kong suggested that most students are insufficiently active during PE periods (Ha et al.: Autonomous motivation predicts moderate-to-vigorous physical activity and psychological well-being in Hong Kong schoolchildren, submitted).

Considering the low levels of PA in many PE lessons and the potential health benefits of PA, interventions are needed to increase students’ accumulation of MVPA during PE. In a recent review and meta-analysis of interventions designed to increase MVPA in PE, Lonsdale and colleagues [6] found that fitness infusion interventions (i.e., the integration of vigorous physical activity into other activities) were effective in increasing students’ PA levels during PE. However, the authors noted the lack of high quality studies evaluating fitness infusion interventions and the need for further work in this area. Therefore, the aim of our study was to design and evaluate the effectiveness of an intervention intended to increase students’ MVPA during PE in Hong Kong.

Researchers have found that there is a decline in PA levels with age in children and adolescents [9], therefore our intervention was specifically aimed at secondary school adolescents. Rope skipping was chosen as an activity to be used as part of our intervention. This activity was chosen for numerous reasons. First, rope skipping is a form of moderate-to-high intensity PA [10], thus can be considered as an appropriate fitness infusion tool. Second, rope skipping is low-cost, and therefore could be promoted to schools and students from lower socioeconomic classes. This is important because studies have found that adolescents of lower, compared with higher, socioeconomic statuses may be less physically active [11]. Therefore, the designed intervention could be applicable to all schools and students. Thirdly, students, particularly girls, found rope skipping to be an enjoyable activity [12]. Finally, Hong Kong is one of the most densely populated cities in the world, and schools typically have little space for students to engage in PA [4]. Rope skipping is an activity which does not require a lot of space, hence is suitable for the specific school environments in Hong Kong [13].

### Motivational determinants of physical activity participation

Although the primary aim of PA promotion interventions are to increase students’ instantaneous MVPA, the long-term effects of the intervention in terms of supporting future PA participation should also be considered. As motivational factors are strong precursors to future behaviors, indicators of these constructs should also be used to evaluate an intervention. Specifically, interventions could be considered as more successful if they facilitate types of motivation that support long-term persistence in PA. According to self-determination theory (SDT) [14], motivation can be broadly classified into autonomous (i.e., engaging in an activity for fun and enjoyment or to attain valued outcomes) and controlled (i.e., avoiding self-guilt or acting under pressure from others) forms of motivation. Researchers have shown that autonomous motivation positively predicted students’ percentage of time spent in MVPA during PE [15,16]. In contrast, time spent in MVPA was either negatively associated with [16], or was unrelated to [15], controlled forms of motivation. As autonomous motivation is related to persistence in an activity and psychological well-being [14], while controlled motivation may be detrimental to long-term engagement, the intervention should be evaluated based on students’ changes in these types of motivation towards PE. A “successful” intervention should not only increase students’ PA levels, but also promote more autonomous forms of motivation. Further, it should not increase students’ controlled motivation. Within SDT, the quality of teacher-student interaction may also support or undermine students’ PA engagement. Specifically, the degree to which teachers are autonomy supportive may affect students’ motivation in PE [17]. Autonomy supportive behaviors include promoting fun and enjoyment in students, or providing meaningful rationales for activities. Perceived autonomy support of teachers was found to be associated with students’ autonomous motivation toward PE [18,19]. In this study, we propose to examine whether perceived autonomy support may be associated with students’ autonomous motivation to PE, and therefore their engagement in PE.

### Objectives of current study

The main objective of the current trial is to examine whether the implementation of a 15-minute rope skipping activity at the start of PE lessons would increase secondary school students’ percentage of time spent in MVPA during their classes. Changes in autonomous and controlled forms of motivation towards PE, as secondary outcomes, will also be used to evaluate the effectiveness of the intervention. The intervention will be implemented by the PE teachers, assisted by rope skipping ambassadors provided to schools [12]. The main roles of ambassadors will be to assist teachers in running the classes, demonstrate rope skipping skills taught by the teacher, and to ensure teachers adhere to the structured plan provided to them during the training. As the intervention will be delivered at the class level, a cluster (i.e., class) randomized controlled trial was considered to be the most appropriate study design. Specifically, the intervention will be implemented in classes assigned to the experimental group for four consecutive lessons. While teachers of classes allocated in the control (i.e., delayed intervention) group will employ usual teaching practices during the experimental period. We hypothesize that students’ autonomous motivation towards PE would predict higher percentages of time spent in MVPA during PE classes. Furthermore, after controlling for students’ autonomous motivation and perceived autonomy support from their teachers, students in the experimental group, compared to those in the control group, will spend higher percentages of time in MVPA during three lessons when the intervention is applied.

## Methods/Design

### Trial design

**Figure 1:**
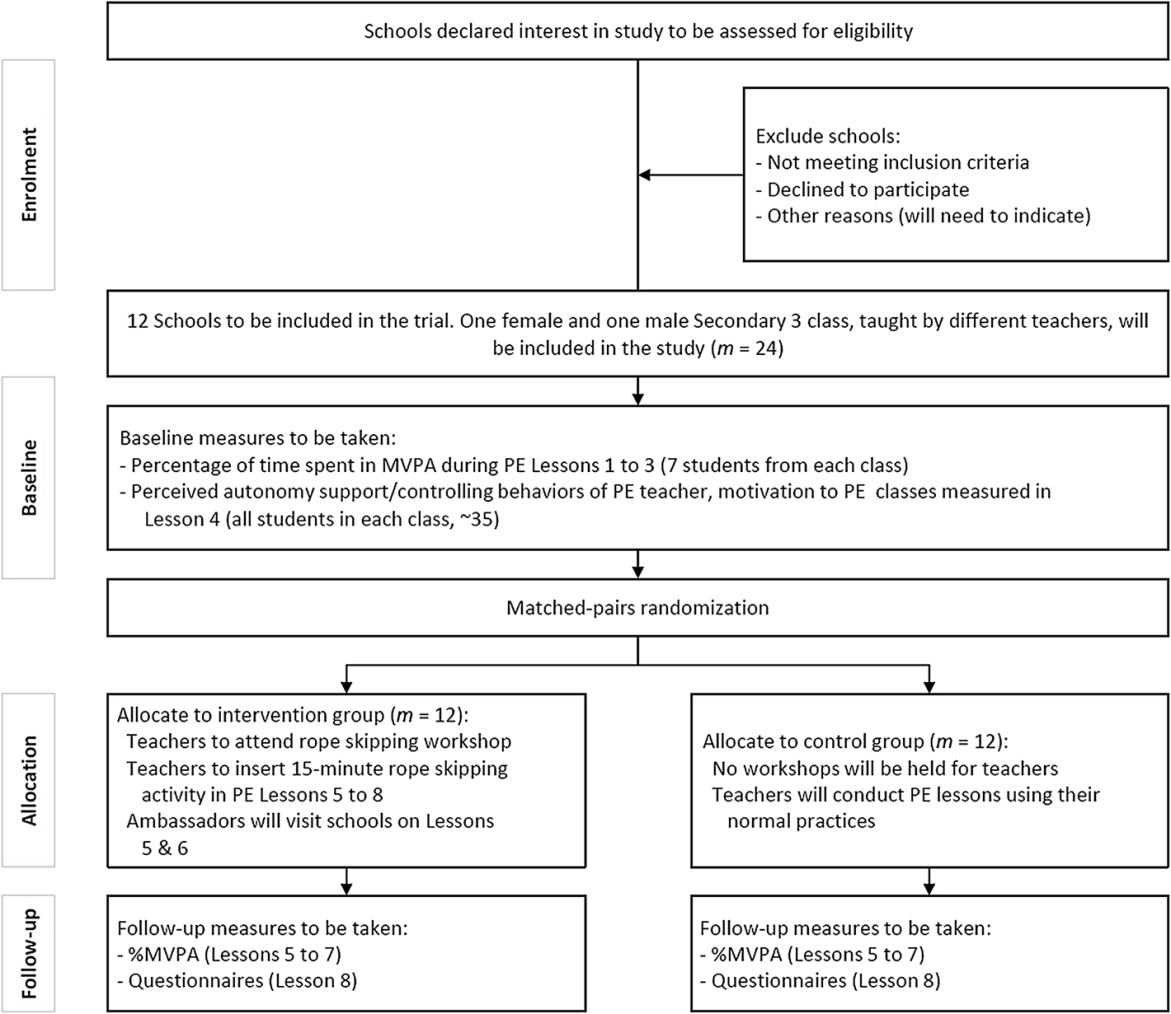
The flow diagram of the study protocol.

**Figure 2:**
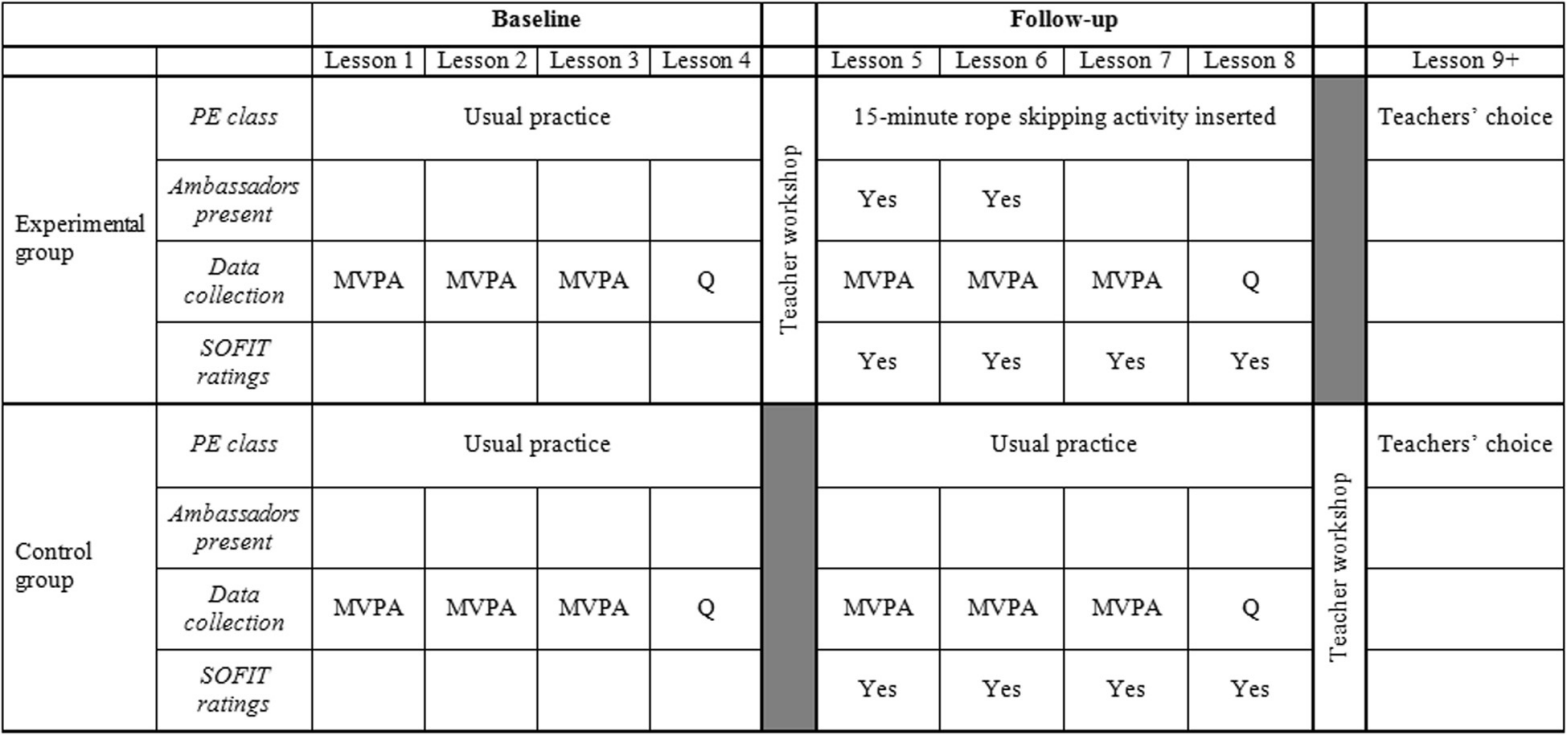
**Timeline of baseline and follow-up data collection.** MVPA = moderate-to-vigorous physical activity measured using accelerometers; Q = questionnaire measures of motivation towards physical education, and perceived teacher autonomy support.

A cluster randomized controlled trial will be conducted to evaluate the impact of the rope skipping intervention on MVPA in PE lessons. A flow diagram and timeline of the design protocol are shown in Figures 1 and 2, respectively. Randomization will be carried out at the class level. Allocation ratio of experimental and control groups will be set at 1:1. Each class will be defined as a separate cluster. All procedures of the study will be carried in compliance with the Helsinki Declaration. Ethic approval for the study protocol was obtained from the Joint Chinese University of Hong Kong-New Territories East Cluster Clinical Research Ethics Committee (Reference: CRE-2013.235).

### Participants

Secondary 3 to 4 students (typically 14 to 15 years old) from local Hong Kong schools (i.e., Cantonese speaking, with vast majority of students being Chinese) will be recruited to take part in the study. All classes included in the trial will be taught by different PE teachers. Students will need to complete the Physical Activity Readiness Questionnaire [20] to assess their physical health and readiness to take part in physical activity. Based on the response to the questionnaire, students having no known undesired physical reactions (e.g., chest pain, dizziness, joint problems) to physical activity will be considered eligible to take part in the current study. Written informed consent will be obtained from participating students and a parent or guardian prior to data collection.

Students will be recruited from schools that meet our eligibility criteria. Specifically, schools will be eligible if (a) they are co-educational (i.e., mixed-sex); (b) they are located in areas of lower-half of the socioeconomic classes within Hong Kong, as defined by the 2011 census [21]; (c) they have a unisex PE lesson policy (applies to most schools in Hong Kong to increase generalizability). Baseline (i.e., pre-randomization) and post-test (i.e., post-randomization) data collection will be conducted during PE lessons. Pre-randomization data collection will begin in September 2013; whereas the post-test data collection will begin in October 2013.

### Intervention

A 4-hour workshop will be provided to teachers of classes allocated to the experimental group. The goal of the workshop is to teach PE teachers basic rope skipping skills, and to demonstrate how the intervention could be delivered in their classes. Rope skipping ambassadors will also attend the workshop to familiarize themselves with the teachers and the structure of the 15-minute activity. The workshop will be led by a professional rope skipping coach (“the coach”), who has experience as a full-time school PE teacher, and therefore understands how typical PE lessons in Hong Kong are structured.

The workshop will consist of four sections. In the first section, teachers will be taught rope skipping techniques. These techniques will be included in the two 15-minute rope skipping activities proposed to teachers. In the second section of the workshop, the coach will demonstrate two 15-minute rope skipping routines that could be implemented by teachers during their lessons in the intervention period. In this session, teachers will be asked to follow the routine led by the coach. The goal of this task is to help teachers understand how the intervention could be implemented, and to experience the intervention from a student’s perspective.

In the third section of the workshop, the coach will discuss with teachers good and poor practice when conducting the intervention, in terms of how to maximize students’ physical activity within the 15-minute rope skipping activity period. The good and bad practices to be highlighted were derived based on discussions between the lead author, a co-author, and the coach. Videos demonstrating these practices will be shown to teachers to assist in the discussion. Videos of good practices will feature the coach teaching one technique by 1) breaking down complex skills (e.g., normal rope skipping) into series of simpler skills (e.g., skipping without the rope); 2) allowing students to adjust to different levels of difficulty; 3) helping students develop the skill by drawing on their previous rope skipping skills and experiences. These practices will encourage students of lower abilities to remain engaged in physical activity. In contrast, the video showing poor teaching practices will feature the coach 1) spending too much time teaching verbally when students stand or sit still; 2) teaching a skill that may be too difficult for most students. The two sets of practices were pilot tested on two groups of three students each. Students who were taught using the good practices exercised in MVPA for 57.5 ± 3.5 percent over a five-minute period. In contrast, students taught using the bad practices spent 20.8 ± 3.6 percent of the five-minute period in MVPA.

In the fourth section of the workshop, the lead author and the rope skipping coach will lead discussions about potential challenges teachers may face when conducting the intervention, and ways to overcome these obstacles. Teachers will be encouraged to contribute to the discussion by reflecting on their experiences in teaching rope skipping or similar activities at their respective schools and during the workshop. They will be encouraged to identify potential barriers and solutions for delivering the intervention in their schools, or how to overcome any minor incidents they can foresee. The goal of the discussion is to ensure all teachers have similar knowledge and expectations for conducting the rope skipping activity, and therefore reduce the discrepancies in the actual implementations of the activity.

The intervention in schools will involve a 15-minute rope skipping activity that is implemented by the class PE teacher at the start of four consecutive PE lessons (during post-test periods). After the rope skipping activity, a usual lesson as run by the PE teacher will be resumed. A four-lesson duration was chosen because, following Hong Kong governmental guidelines [22], the typical length of teaching an activity during PE (i.e., a unit) is four lessons. In the first two lessons of the four-lesson post-test period, a skipping ambassador [12] will be sent to each class to demonstrate rope skipping skills, and assist the teacher in running the 15-minute activity and instructing students. In the second to lessons, the teacher will lead the rope skipping activity. A wait list control group will be adopted in the study. That is, classes allocated in the control group will receive the intervention after the end of the study period.

### Fidelity of intervention

Teachers in the experimental group will be asked not to discuss the content of the workshop with control group teachers whom they may encounter. To ensure intervention fidelity (experimental group) and test for any contamination effects (control group), raters blinded to study hypotheses will observe all PE lessons run by each teacher (including those assisted by the skipping ambassadors) during the study period. The System for Observing Fitness Instruction Time (SOFIT) [23,24] will be used by the rater to record the activities students engaged in during each lesson, the lesson context, and teacher involvement. Additional to the SOFIT protocol, the raters will also need to report whether students were engaging in activities related to rope skipping. Raters will record the specific start and finish times of rope skipping activity. They will be asked to mark the time (to the closest second) based on a web-based clock showing the official time of Hong Kong, provided by the Hong Kong Observatory. The computer used to initialize the accelerometers will also be synchronized to the same time. Raters without an internet-enabled mobile phone will need to report the offset of their time-telling device with the official time when they return the accelerometers and records to the investigators.

Raters will be university students from a Hong Kong university. They will receive a 2-hour training session led by the lead author and a co-author, based on the SOFIT training manual [24]. Video recordings of PE lessons, for classes in both experimental and control groups, during the intervention period will be taken. Each class will be video-recorded at least once during the post-test period. At least one rating made by each rater will be cross-checked by an investigator of the study using the recorded videos clips. These ratings will be used to examine the inter-rater/class reliability.

### Outcomes

#### Primary outcome

The primary outcome of the study is the proportion of PE lesson students spend in MVPA. This will be measured using ActiGraph (Pensacola, Florida) GT3X + accelerometers. MVPA will be defined using Evenson and colleagues’ criteria [25], and 1-second epochs will be used [26]. Evenson et al.’s cutoff values were chosen because they were found to be more accurate than other commonly used cut points [27]. To test the validity and sensitivity of the devices of capturing non-lateral movements such as rope skipping, we invited 12 boys and girls with varying rope skipping experience perform rope skipping tricks (in 1-minute bouts with at least one minute rest in between) that will be included in the intervention while wearing accelerometers. For all skippers and for all tricks tested, the activity was classified as MVPA based on Evenson et al.’s cut points. In our formal trial, accelerometry measurements will be taken during the first three lessons at baseline (Lessons 1 to 3; see Figure 2). MVPA will be measured for the same sets of seven students in each class throughout the study. These students will be randomly selected by the investigators from class lists (not containing names of students) provided by teachers using a random number generator. The mean of each student’s proportion of time spent in MVPA during these three classes will be used as his or her baseline. The primary outcome will pertain to the individual level and will not be aggregated within the cluster.

After baseline assessments, classes will be randomized into either the experimental or the control group. Teachers of classes in the experimental group will be invited to attend the teacher workshop after Lesson 4, and before Lesson 5. Following the workshop, these teachers will implement the intervention in their class for four consecutive PE lessons (Lessons 5 to 8). For schools in the control group, teachers will conduct classes using their usual methods, which are not expected to include rope skipping. Post-test accelerometry measures will be taken during Lessons 5 to 7. The mean of each student’s MVPA percentages taken from these three classes will be used as follow-up scores. Similarly, this measure pertains to the individual level.

#### Secondary outcomes

Secondary outcomes include students’ time spent in sedentary, light, moderate, and vigorous physical activity as measured by accelerometry, and defined using Evenson et al.’s [25] cut-points. Perception of autonomy support provided by their teachers during the last four PE lessons, and their motivation (autonomous and controlled) during the last four PE lessons were also measured as secondary outcomes. Psychological variables will be measured using self-reported questionnaires administered using pen and paper at the end of Lesson 4 (baseline) and Lesson 8 (post-test). Specifically, the 6-item version of the Learning Climate Questionnaire [28] will be used to measure students’ perceived autonomy support provided by their teachers. The original scale in English was translated into Chinese using a back-translation protocol. As the translated scale has not previously been used, factorial validity of responses will be examined before the main analyses are conducted. In terms of motivation orientations, the Chinese Perceived Locus of Causality Questionnaire (PLOCQ) [29] will be used to measure autonomous and controlled motivation. Evidence of validity and reliability of the scale scores has been shown by Lonsdale et al. [29]. As motivational factors are likely to differ across individuals, students’ motivation towards PE was considered as a variable at the individual level. In contrast, as perceived autonomy support is referenced to the same teacher, the mean scores within each cluster will be aggregated to form an indicator representing this construct at the cluster level.

### Sample size

Sample sizes were calculated based on the effect sizes calculated from Lonsdale et al.’s [6] systematic review and meta-analysis. In their study, Lonsdale et al. found that the standardized difference, or Cohen’s *d*, of fitness infusion interventions implemented within PE classes to be 1.4. Adopting a conservative approach, we based our power calculation on a projected effect of *d* = 1.0. The required sample size was calculated using GPower 3.1, with alpha level set to .05, and power at .80. The required sample size for a two-tailed independent sample t-test was found to be 34 students.

To account for the clustering nature of the data, the required sample size of 34 was adjusted by design effect [30] of 1 + (*m*-1)ρ, where *m* is the sample size of each cluster and ρ is the intraclass coefficient (ICC). In order to limit the disruptions to classes when distributing accelerometers, we decided to administer the device to seven students in each class. Aelterman et al. [15] found in their study that the ICC of MVPA between classes was .63. Using these estimates, the required correction factor is 1 + (7–1).63 = 4.78, meaning that the total required sample size is 163 (4.78 × 34 students). Therefore, 24 classes will need to be recruited for the study (24 classes × 7 students = 168 students).

### Randomization

#### Sequence generation

Randomization and allocation to experimental and control groups will be conducted after baseline measures are taken. Matched-pairs cluster randomization will be used. That is, randomization and group allocation will be conducted at the cluster (i.e., class) level, Specifically, participating classes will be matched in comparable pairs in terms of 1) class gender, 2) students’ percentage of time spent in MVPA measured at baseline, and 3) whether rope skipping is included in the school’s original PE curriculum. Baseline measures will be dummy coded and sent to a statistician outside the research group, who will then randomize and allocate classes in experimental and control groups. This person will have no information regarding the participating schools or classes. The statistician will match the pairs and randomize classes into experimental and control groups using a random number generator. After randomization, teachers will be told the group allocation of their classes. The allocation mechanism, however, will be concealed to them.

#### Blinding

Teachers will not be blinded to group assignment, as they will attend rope skipping workshops and need to implement the intervention. Participants will be notified about the existence of randomization and group allocation procedures, but they will be blinded to the study hypotheses. All research assistants and raters for the SOFIT protocol will also be blinded to the specific hypothesis of the study. They will not be specifically told about the randomization and allocation of groups, but we will not consider them to be completely blinded to these as they may rate classes from both experimental and control groups and hence may be able to ascertain that some classes are assigned specific tasks.

### Statistical methods

Multilevel modeling will be used to account for the effects of participant clustering. Specifically, a three-level (time within student within class) model will be examined. For our primary outcome of students’ percentage time spent in MVPA, the effects of group allocation and student gender on percentages of time spent in MVPA will be examined using a multilevel regression model. The group × gender interaction effect will also be examined to determine whether the effect of the intervention is homogeneous across genders. Further, we will also examine a model by adding perceived autonomy support, autonomous and controlled motivation as potential confounders of the outcome. Two-tailed tests with an alpha level of .05 will be used to determine the significance of all results. To examine whether the intervention would lead to changes in motivation variables and perceived autonomy support by teachers, these variables will be regressed on group, gender, and group × gender in an additional series of three-level regression analyses. Statistical analyses of the study will be conducted by a statistician blinded to the hypotheses of the study and to each class’ allocation to the experimental or control condition.

### Teacher and student interviews

After the collection of quantitative data for the study, all (i.e., 12) teachers in the experimental group will be interviewed individually. The students (seven per class) in the experimental group who wore accelerometers during the study will be invited to take part in focus group interviews. Interviewees will be asked their views on the intervention, such as whether they, or felt other students, enjoyed the new approach. Teachers and students will also be asked whether they felt the intervention was successful in increasing students’ activity levels. We will seek their opinion in terms of whether the intervention could be promoted to other schools, and how it could be improved.

## Discussion

Researchers have shown that most students engage in insufficient MVPA during PE classes [8]. To address this issue in space-confined school environments, such as those in Hong Kong, we designed an intervention based on rope skipping to increase students’ MVPA during PE lessons. The protocol described above provided details of how the intervention will be implemented and evaluated. The evaluation of this protocol is important because although students are expected to spend a high percentage of time in MVPA during the 15-minute rope skipping activity, it remains to be seen if the intervention increases the overall proportion of lesson time spent in MVPA compared to existing lessons. For instance, the contents of existing PE lessons may provide students with similar exposures to MVPA. If student MVPA levels increased during the 15-minute period, compensation effects may exist such that students may be less active during portion of the lessons [31]. Further, the current protocol allows us to explore whether the intervention effect is homogeneous across boys and girls. This is important because boys and girls may have different preferences towards the type of activity or game played [32]; therefore, examining the interaction effect on boys and girls separately may assist in future interventions or school policies aimed at increasing students’ activity levels.

Based on our knowledge, this is the first intervention designed specifically to increase students’ MVPA during PE lessons in space confined environments. Apart from MVPA, we proposed to simultaneously examine the effects of the intervention on students’ autonomous and controlled motivation towards PE, as specified by SDT [14]. Based on the theory, these outcomes have important ramifications to students’ future engagement in PA, and also their physical and psychological well- and ill-being. Accordingly, we hypothesize that the intervention would increase students’ MVPA during PE, and also support more adaptive forms of motivation. In this study we also proposed to measure the perceived autonomy support and control by PE teachers. Within SDT, these perceptions are important predictors of students’ motivation towards PE, and in turn the amount of MVPA they engage in class. Our study will provide preliminary evidence on the relation between perceived teacher behaviors and students’ objectively measured activity levels. Further, our results could inform future research directions in terms of methods to further improve PE intervention effectiveness. For instance, future work could investigate whether the intervention could also further enhance students’ autonomous motivation if teachers were given training to be more autonomy supportive [33]. In conclusion, the results from our study may advance both teaching practices and theory alike.

## Competing interests

The authors declare that they have no competing interests.

## Authors’ contributions

AH conceived the study, provided advice on the design and edited the manuscript. JN provided advice on the design of the study and wrote the initial draft of the manuscript. CL and DL provided advice on study design and edited the manuscript. All authors read and approved the final manuscript.

## Pre-publication history

The pre-publication history for this paper can be accessed here:

http://www.biomedcentral.com/1471-2458/14/535/prepub
